# Supplementary figures and images for: The dental triage method at Rothschild Hospital during the first lockdown due to the COVID-19 pandemic
Source: PLoS One. 2023 Feb 8;18(2):e0281390. doi: 10.1371/journal.pone.0281390 (PMC9907804; doi:10.1371/journal.pone.0281390)

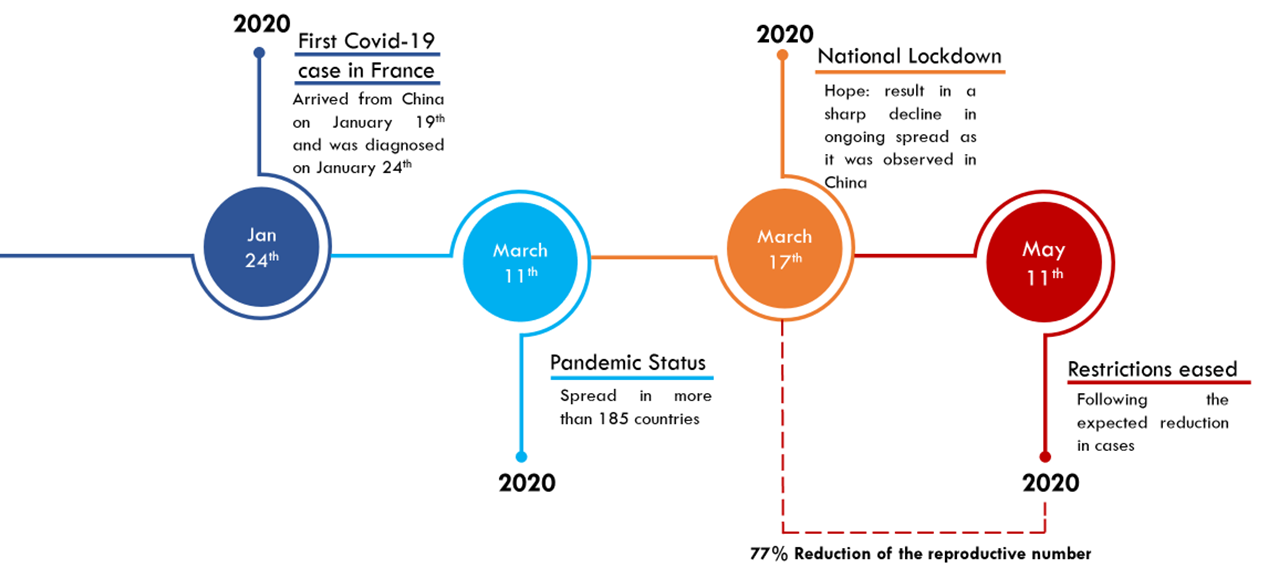

Supplement: S1 Fig — (TIF) [file pone.0281390.s001.tif]

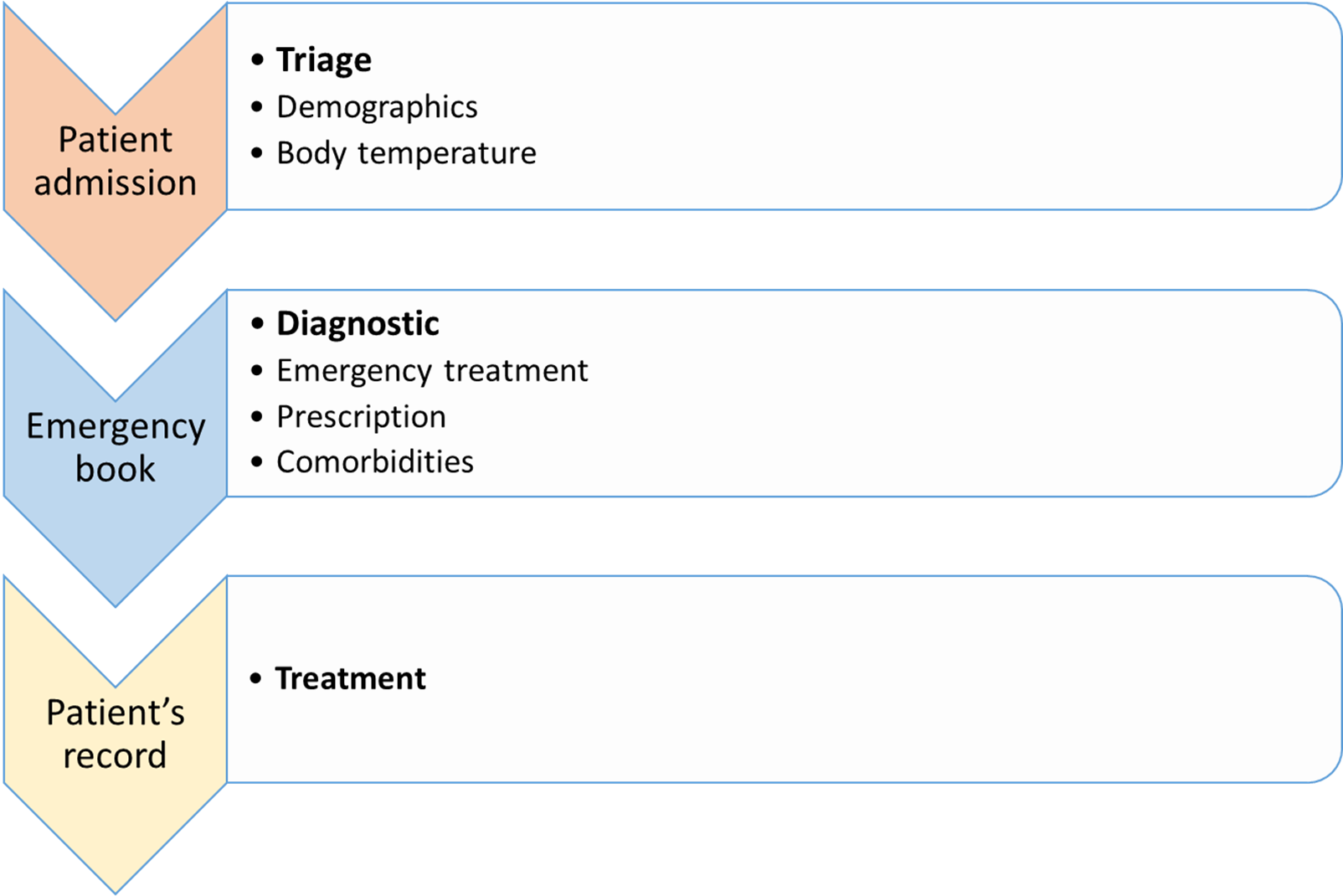

Supplement: S2 Fig — (TIF) [file pone.0281390.s002.tif]

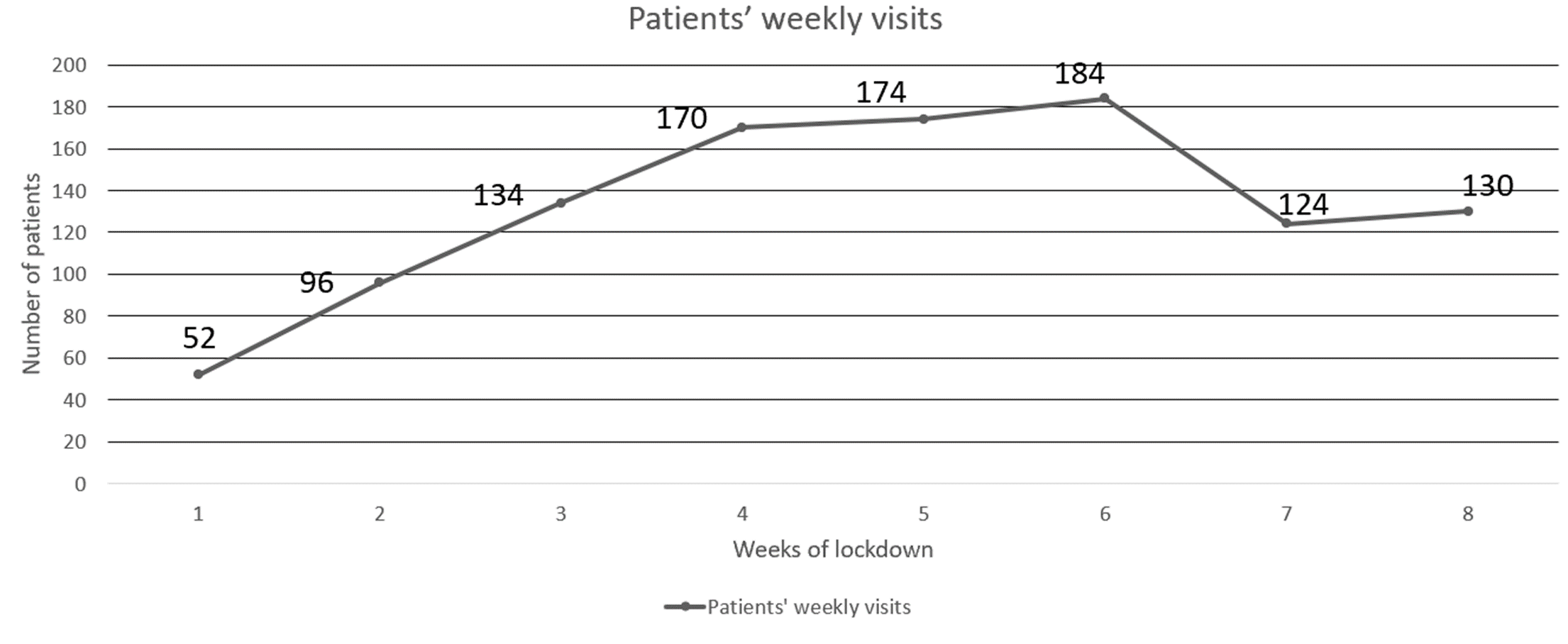

Supplement: S3 Fig — (TIF) [file pone.0281390.s003.tif]

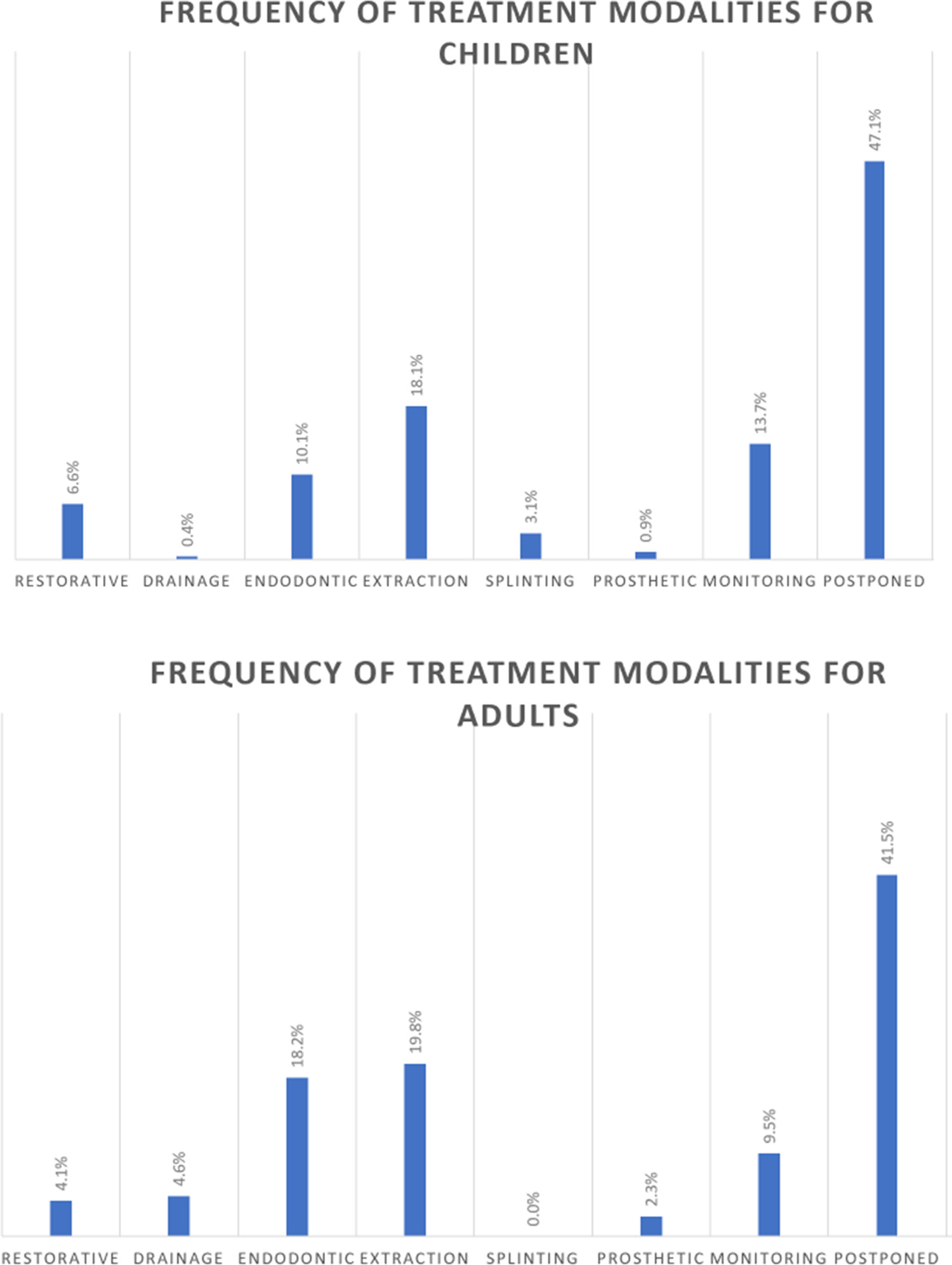

Supplement: S4 Fig — Treatment modalities for children (A) and adults (B). (TIF) [file pone.0281390.s004.tif]
