# Supplementary material for: The dental triage method at Rothschild Hospital during the first lockdown due to the COVID-19 pandemic
Source: PLoS One. 2023 Feb 8;18(2):e0281390. doi: 10.1371/journal.pone.0281390 (PMC9907804; doi:10.1371/journal.pone.0281390)
Supplement: S2 Table — Details of the different types of diagnoses are given for each category. (PDF) [file pone.0281390.s006.pdf]

Supplementary Table 2: Distribution of the number of subjects among the 4 groups analyzed according to triage or diagnosis and their difference in adults. Details of the different types of diagnoses are given for each category.

Emergency groups	*Group 1*<br><br>*Endodontics; Periodontics; Infectious symptoms*	*Group 2*<br><br>*Prosthetic*	*Group 3*<br><br>*Trauma*	*Group 4*<br><br>*Others*
Triage n (%)	*533 (87)*<br><br>*Periodontal; endodontic; cellulitis, pain, abscess*	*9 (2)*<br><br>*Prosthetic*	*19 (3)*<br><br>*Trauma*	*49 (8)*<br><br>*Other*
Diagnosis n (%)	*509 (83.4)*<br><br>*Food impaction; ANUG;*<br><br>*Gingivitis; hopeless tooth prognosis; necrosis; pulpitis; acute apical periodontitis; cellulitis; abscess; pericoronitis*	*20 (3.3)*<br><br>*Prosthetic*	*57 (9.3)*<br><br>*Trauma; fracture; luxation*	*24 (3.9)*<br><br>*Eruption; adenopathy*
Triage vs. diagnosis difference n (%)	*24 (5)*	*-11 (55)*	*-38 (67)*	*25 (51)*
